# Epigenetic Mechanisms of Paternal Stress in Offspring Development and Diseases

**DOI:** 10.1155/2021/6632719

**Published:** 2021-01-19

**Authors:** Xingyun Xu, Zhigang Miao, Miao Sun, Bo Wan

**Affiliations:** ^1^Institute for Fetology, The First Affiliated Hospital of Soochow University, Suzhou City, China; ^2^Institute of Neuroscience, Soochow University, Suzhou City, China

## Abstract

The major biological function of the sperm cell is to transmit the paternal genetic and epigenetic information to the embryo as well as the following offspring. Sperm has a unique epigenome. An increasing body of epidemiological study supports that paternal stress induced by environmental exposures and lifestyle can modulate the sperm epigenome (including histone modification, DNA methylation, and noncoding RNA expression), sperm-egg fusion, embryo development, and offspring health. Based on the existing literature, we have summarized the paternal exposure on sperm epigenome along with the representative phenotypes of offspring and the possible mechanism involved.

## 1. Introduction

Genetic inheritance occurs due to DNA and gene being passed from parents to their offspring. Compared with genetics, epigenetics is the alteration in heritable traits in gene expression without altering DNA sequence. In recent years, multiple lines of evidences have pointed that epigenetic alterations affected by environmental factors are critical in complex chronic disease, but the role of epigenetics may be underestimated. Epigenetic alterations could also lead to a chain reaction of generations like the genetics do.

David Barker has put forward the hypothesis that the fetus and infants are easily affected by the adverse environment intrauterine or during the early developmental stage, and it could increase the susceptibility of the adult offspring to cardiometabolic diseases. That is, “the fetal origins of adult disease (FOAD)” [1]. With the deepening of understanding of FOAD, people deem that early life adverse environmental exposures threaten the health of the subsequent stages, not only the fetal stages but also the male and female gametes (sperm and ovum), zygote, and early embryos [2]. The current situation of both parents exposed to environmental factors (diet, mental pressure, poisons, pollutants, etc.) will impact offspring health in a nongenetic way (including histone modification, DNA methylation, and noncoding RNA). These environment factors trigger epigenetic reprogramming of parental genome, without altering DNA sequence [3–7]. Previous studies have mainly focused on the epigenetics and pregnancy effects of maternal exposure. Emerging animal and human studies provide evidences of the link between paternal experiences and heritable epigenetic programming.

Sperm is similar to tadpoles in shape and carries the paternal genetic material. The principal function of the sperm is to complete fertilization by combination with an ovum and deliver paternal genetic material into the oocyte to form a zygote. Spermatocytes are differentiated into haploid genomic cells in the spermatogenesis process. Mammalian sperm cells are characterized with compact nuclei and movable flagella; these special physiological structures contribute to the integrity of the genome [8]. Plenty of data supports that environmental exposures and paternal lifestyle can alter sperm epigenome, and affect embryo development and offspring health after fertilization.

The integrity of sperm DNA contributes to the optimal embryo development and offspring health. However, it is clear that the highly dynamic and environment-sensitive epigenetic information of the paternal nucleus also plays a critical role in the offspring health.

In this review, we focus on sperm epigenome and summarize the paternal stress exposures on sperm epigenome along with the representative phenotypes of offspring and the possible mechanism involved.

## 2. Intergenerational Inheritance Caused by Paternal Stress in Epidemiological Study

Intergenerational epigenetic inheritance refers to the transmission of epigenetic information through the germline and persisting in subsequent generations. Numerous examples of intergenerational epigenetic inheritance in humans have been reported. For instance, the offspring of fathers who had experienced the Holocaust war during the Khmer Rouge period in Cambodia were more likely to suffer from anxiety and depression, and the children of Australian veterans in the Vietnam War were also more likely to commit suicide [9, 10]. Smoking in young men seemed to affect offspring respiratory health born years later. Epidemiological study showed that fathers started smoking before age 15, and even if they stopped smoking for more than five years before fertilization, the risk of asthma in offspring was still high [11]. Endocrine disruptors (EDCs) cause a series of adverse reproductive health consequences by interfering with hormone function. Phthalate is one of the EDCs used in a variety of daily commercial products. Among 50 couples with in vitro fertilization (IVF), the concentration of phthalate in the father's urine was inversely proportional to the quality of blastocyst. The study indicated that the physiological balance of the father before fertilization directly affects the reproductive ability [12]. There was evidence that older fathers were associated with several neurological disorders in their child. The risk of a child being autistic increased dramatically with the father's age: the incidence of autism in offspring of men (aged ≥50 years) was 2.2 times higher than offspring of younger men (aged ≤29 years) [13]. There was a significant negative correlation between the age of the father at children's birth and children's social dysfunction of early deviant behavior [14]. The clinical data supported the paternal alcohol consumption was closely related to the behavior of the offspring, the social psychological abnormality, the children's neurocognitive development disorder, and the physiological defects (such as the decrease of intracranial volume, the change of electroencephalogram, and the difference of neuroimaging) [15]. A new research has discovered that a grandfather's food supply was only related to the death rate of the grandson, while a grandmother's food supply was only related to the death rate of the granddaughter [16]. All those epidemiological studies indicate that sperm cells can be easily disturbed by the environment stress and result in the increasing of the susceptibility of offspring to disease.

## 3. Epigenetics of the Sperm

The sperm nucleus has a higher order of packaging structure and is about six times smaller than an interphase somatic cell. During spermiogenesis and fertilization, histone-to-protamine transition involves in chromatin remodeling. Epigenetic factors, such as chromatin, DNA, chromatin proteins, and associated marks as well as sperm-derived RNA (in particular sncRNAs), constitute a specific epigenetic landscape of sperm, which plays a critical role in sperm chromatin remodeling and zygote, embryonic, fetal, and offspring development.

### 3.1. Expression of Histones in Mature Sperm and Participation in Chromatin Remodeling after Fertilization

Sperm is formed in the testis, a haploid genomic cell with specific biological functions for fertilization [17]. During spermatogenesis, the haploid genome undergoes chromatin reorganization through meiosis and DNA condensation [18]. To complete chromatin remodeling, in the late stage of spermatogenesis, testicular specific variants are required to replace somatic linker histones [19]. In addition, the highly positively charged protamine and the phosphodiester skeleton of the DNA in the sperm are entangled into a ring in a nonspecific manner, making the sperm genome inactive. After sperm-egg fusion, the protamine is removed and the genome is activated [20]. Sperm DNA wraps around protamine to achieve a highly compact chromosomal structure, which is different from the nucleosomes of somatic cells and is concentrated at least 6 times [21]. Histone H4 superacetylation replaces histones with protamine, providing a loose chromatin conformation and completing the spermatogenesis process [22–24]. Nevertheless, protamine does not completely replace histones, and 5%-15% of histones still exist on chromatin [25–27]. Do the remaining histones in mature sperm play other important biological functions or are they due to incomplete replacement of protamine? Studies revealed that the remaining histones were not randomly distributed in the chromosome but inserted into specific regions [28]. This indicates that histones have more function and provide new insights into the interaction between germline genetic and epigenetic functions. It is agreed that protamine does not involve epigenetic modification, and epigenetic modification regulates posttranscriptional modification through histones.

During fertilization, the sperm needs to be integrated into the oocyte. At this time, the sperm and oocyte genome are in a state of inactivation, and the germ cells develop into totipotent embryos through “zygotic genome activation” [29, 30]. The zygotic genome is activated by protamine-to-histone exchange through the incorporation of maternal histone variants (H3.3, H1FOO, microH2A, and H2A.Z) [31–34]. Histone variants mediate chromatin remodeling after fertilization [35]. During the first mitosis of the sésame Drosophila zygote, synthetic histone variants H3.3 is expressed on the paternal chromosome, and it is involved in the formation of paternal chromatin [35].

Sperm histones participate in the gene profile expression of embryos [36]. The retained histones are involved in the transmission of information across generations. In this way, the offspring continue this “epigenetic modification variation.”

### 3.2. Genomic Imprinting

Genomic imprinting refers to the genetic process wherein a gene is differentially expressed depending on whether it has been inherited from the mother or from the father. Diploid genes silence paternal-derived chromosomes through “maternal suppression,” thereby transforming into haploid expression, and the genes involved are imprinted genes [37]. More than 70 imprinted genes have been identified in mammals [38]. Genomic imprinting is regulated by DNA methylation [39]. After fertilization, the oocytes have two different epigenetic pronuclei from the male parent and the maternal parent. The paternal genome is rapidly and actively demethylated in 6-8 hours after fertilization [40]. The maternal pronucleus is gradually passively demethylated during the subsequent division [41]. Despite the first large-scale wave of demethylation, imprinted genes can be protected from demethylation, and the imprint of the parent can be “inherited” [42, 43]. These imprint marks were erased in the primordial germ cells (PGC) in E10.5-E12.5 embryonic days post coitum (dpc), which is the second wave of demethylation [44]. After two waves of global demethylation, the PGC genome at E13.5 dpc undergoes remethylation at specific regions for building an inherited imprinting map in generation [45]. It is worth noting that certain elements can escape these two major demethylation events and form heritable imprints in the germline [44].

Long-term exposure to stress induces germ cells to produce “markers,” reprogram genetic information, and affect the neurodevelopment of offspring [46–49]. The factors affecting maternal genetic information are complex, changeable, and multistage, The whole system of pregnant women, fetuses, and newborns is interrelated, and it is regulated and mediated by many factors such as uterine microenvironment, breastfeeding, and maternal behavior [50]. Sperm, as the male parent's genetic information, is relatively straightforward and single.

## 4. Characteristics of Paternal Stress

Many studies based on paternal stress transmission have testified that the age of paternal stress does influence in this transmission. The baseline anxiety of offspring in the stress state during early life was lower than that of offspring in adolescence or adulthood [51–53]. In addition, the type of stress, the duration of stress, and the physiological state of sperm during the stress period are all factors that cannot be ignored [54]. In short, stress can be divided into social and asocial stress. For social stress, the exposure of the father in different periods determines the anxiety transmission of offspring [54]. The determinants of unhealthy phenotypes in offspring induced by paternal stress need to be further studied so as to be clearer.

## 5. Maternal Influence on Paternal Germline Transmission

The influence of maternal stress on children's health is more complicated than that of paternal stress. It can be affected in several stages, from prepregnancy, pregnancy, to lactation. It is obvious that the various behaviors and physiological conditions of mothers affect offspring development. Maternal postpartum stress and changes of the uterus can alter offspring intergenerational epigenetics caused by paternal effect [51, 55]. It is worth considering whether maternal and paternal effects on offspring development will interact. Here, we explored the role of matrilines in the transmission of paternal effects to offspring.

A physiological phenomenon in which the appearance, physiological characteristics, and production performance of offspring are directly affected by their mother is called maternal effects. According to the change of environment, the mother has different investment offsets in order to improve the survival of offspring. Female zebra finches invest differentially in offspring developmental traits (egg resources) in response to attractive or unattractive males. Female birds generally invest more into reproduction when paired with attractive males, both in terms of egg size and number as well as food provisioning [56]. What is interesting is that this distribution of the parturient is a compensation for reproductive defects, and more behavioral care is given to the poor-quality male offspring [57, 58]. It has been reported that maternal care behavior is related to paternal anxiety-like behavior induced by isolated condition [59]. Using embryo transfer technology, it was proved that sperm of adult food-restricted male mice could affect the development of offspring. In a natural mating environment, females who mate with food-restricted males show increased prenatal and postnatal care, reversing phenotypes observed under embryo transfer conditions [60]. From the above evidence, we know that maternal effect can modulate the process of the paternal germline transmission to affect the development of offspring through sperm. The phenotype of offspring is the embodiment of a comprehensive function.

## 6. Mechanisms of Intergenerational and Transgenerational Epigenetic Inheritance

At the molecular level, any alteration in gene expression produces different phenotypes without involving DNA sequence, could be heritable, which are considered epigenetic inheritance [61]. At present, the main contents of epigenetic research include DNA modification, histone modification, and RNA and RNA-associated modification. The connection between the laboratory studies of transgenerational epigenetic inheritance and the evolutionary processes occurring in natural populations were confirmed [62]. The DNA of sperm carries all the genetic information of the father, and the nucleotide sequence change of DNA affects the phenotype of generations. The hypothesis of epigenetic transgenerational inheritance of disease and phenotypic variation induced by environment has aroused controversies. However, ample evidences have shown that DNA methylation modification has a profound impact on the regulation of cell function and development [63].

**Figure 1:**
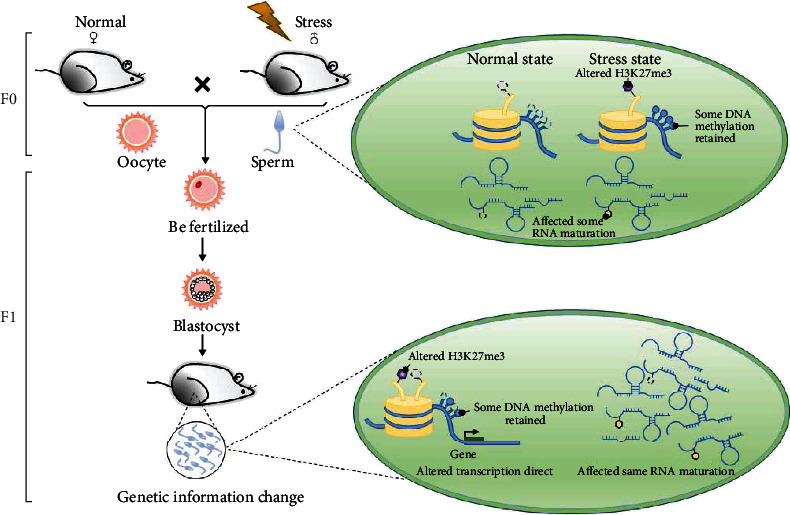
The genetic information of the sperm changes after stress. Under stress, histone H3K27me3 modification, DNA methylation, and RNA maturation were affected in the F0 sperm cells. Some of these modifications can “continue” in the zygote and “inherit” this “marker” to the male F1.

Epigenetic mechanisms are significant for the establishment and maintenance of cell identity. Development origin and tissue microenvironment determine the actual epigenetic state of each genome region. Newborn skin melanocytes and fibroblasts show distinct epigenetic “markers.” Compared with melanocytes and fibroblasts from the same newborn skin, cells from the same region in the ectoderm, such as keratinocytes, breast cavity, and myoepithelial cells, had more epigenetic “markers” [64]. In the process of development, individuals have to undergo multiple generation and elimination of epigenetic “markers.” Among them, DNA methylation, histone modification, and small noncoding RNAs are used to alter the epigenetic “markers” [65]. The proposed mechanism by which environmental exposures are linked to disease phenotypes in offspring is intergenerational and transgenerational epigenetic inheritance (Figure 1 and Table 1).

### 6.1. DNA Methylation

One of the key mechanisms of epigenetic gene regulation is DNA methylation. DNA methylation is the selective binding of cytosine bases of two CG nucleotides in DNA chain to a methyl group in the form of covalent bond. DNA methylation is modulated by DNA methyltransferases (DNMT1, DNMT2, DNMT3A, and DNMT3B) and ten-eleven translocation (TET) methylcytosine dioxygenases (TET1, TET2, and TET3), as well as the thymine-DNA-glycosylase (TDG) and the DNA base excision repair (BER). This modification is most frequently found in the context of CpG dinucleotides and stable during replication and cell cycle arrest [78]. The presence of DNA methylation in gene promoters and enhancers regulate gene expression, likely through alterations of the local DNA structure and modulations of the binding between specific transcription factor and cis-regulatory element. This modification in the 3′ untranslated region also plays a role in regulation of protein expression via interaction with miRNA [79].

DNA methylation provides a molecular memory dedicated to the transcription process in mammalian development. The first demethylation occurs during the formation of primordial germ cells in the embryonic stage, in which DNA methylation originated from parents is eliminated and reestablished during the maturation of germ cells; the second extensive demethylation occurs during the fertilization process, after the formation of oosperm, and in the early stage of embryo cleavage, extensive demethylation occurred in sperm and ovum, respectively, which produces new methylation characteristics [80]. The effect of epigenetic modification on phenotype may escape the epigenetic reprogramming at least twice, and the epigenetic modification is not completely erased, so the offspring can “inherit” the paternal phenotype [81, 82].

Intergenerational genetic information is mainly stored in the sperm of the father. It is diverse in the epigenetic mechanism of the offspring. Among them, DNA methylation is the most thorough. Abundant evidences show that DNA methylation can regulate gene expression through altering chromatin modification, DNA conformation, stability, and the way of interaction with proteins [83]. In 2005, a research team at Washington State University discovered that sperm methylation is a potential cause of transrepresentative changes. The generation males (F1) of pregnant rats (F0) are exposed to endocrine disruptors, and the generation males (F2-F4) bred which form his mating with untreated female rats had increased spermatogenic cell apoptosis and decreased sperm count. Many genes of sperm in the epididymis had undergone methylation changes, such as the CpG island of the lysophospholipase gene (LPLase). It did not affect the F2 generation males bred from F1 female and control male [66]. McCarthy et al. found that nicotine exposure in male mice for three months significantly increased the DNA methylation of the dopamine receptor gene promoter in sperm and furtherly decreased expression of dopamine receptors in the striatum of male adult offspring, and there was an imbalance in brain monoamine levels. Nicotine exposure of male mice resulted in behavioral disorders and changed spontaneous motor ability, attention, and reverse learning ability in multiple generations of descendants [67]. The offspring of male mice which underwent postnatal maternal separation have altered brain plasticity and cognitive functions. The expression of brain-specific gamma isoform of protein kinase C (*Prkcc*) was reduced in the offspring, and DNA methylation at its promoter is altered both in the hippocampus of the offspring and the sperm of fathers. It provides a reasonable mechanism for intergenerational transmission [68]. Epigenetic modification, especially DNA methylation, plays an important role in determining the differentiation potential of mammalian cells and ensuring the normal development. The methylation changes of a specific gene in the parent sperm cannot directly lead to the defective phenotype of the offspring, but it is undeniable that the methylation of CpG islands affects a series of cascade gene expressions, resulting in abnormal gene crossover network expression.

### 6.2. Histone Modification

Nucleosome in sperm affects epigenetics. In mammalian spermatogenesis, most histones are replaced by protamine. However, about 10% of histones are still left to assemble sperm nucleosome. There are different opinions on whether histones are distributed in the gene coding region or gene barren region. In recent years, it has been reported that the retained histones are distributed in the promoter regions and the distal intergenic regions of specific genes [84, 85]. This special distribution affects sperm genome epigenetic modification. Sperm with abnormal chromatin packaging has a low probability of successful insemination [86]. The nucleosomes retained in sperm genome were rich in gene clusters related to development, such as imprinted gene clusters, microRNA clusters, HOX gene clusters, and independent promoters of development transcription and signal transduction factors. The nucleosomes were modified by methylation and acetylation and affected the development of future generations [87].

Sperm histones are involved in chromatin conformation and gene expression through various posttranslational modifications. Lysine residues are the most common acceptor sites of methylation, acetylation, ubiquitination, crotonylation [88], ADP-ribosylation [89], and lactoylation [90]. The amino terminus of the four core histones (H2A, H2B, H3, and H4) stores a rich source of genetic information. The residues are subjected to enzymatic reactions to produce posttranslational modifications, which transmit epigenetic information transgenerationally. H3 at lysine 4 (K4) is specifically methylated by Set9, which is related to the activation of gene transcription [91]. H3 at lysine 9 is selectively methylated by SUV39H and silencing gene expression [92]. Interestingly, histone lysine methylation inhibits its deacetylation. K4 methylation strongly blocks the binding of H3 residues to deacetylase (NuRD) [93]. In vitro kinase experiments proved that H3 K9 dimethylation and H3 Ser10 phosphorylation seriously impair the catalytic ability of SUV39H enzyme [92].

There is ample evidence that sperm histone modification affects the health and phenotype of offspring. Kimmins et al. used a genetically transgenic mouse model to confirm that H3K4me3 was significantly upregulated in mouse sperm overexpressing the demethylase Kdm1a and that developmental genes were highly enriched in target difference analysis [69, 70]. H3K4me3 in the sperm of transgenic mice escaped the epigenetic reprogramming of the fertilized egg [69]. The male offspring with cocaine infusions increased the expression of Bdnf and the acetylation of H3 histone in the cortex, resulting in cocaine resistance and enhancing the binding of acetylated H3 histone to BDNF promoter in cocaine paternal sperm [71]. The ancestral history of liver injury inhibited liver fibrosis in male offspring by increasing the accumulation of sperm histone variants H2A. Z and H3K27me3 at peroxisome proliferator-activated receptor *γ* (PPAR-*γ*) promoter. PPAR-*γ* negatively regulates the activation of fibroblasts and inhibits liver fibrosis in male offspring [72]. Modification of sperm histones may influence the large-scale demethylation wave after fertilization, altering the expression of offspring genes.

### 6.3. Small Noncoding RNAs

It is agreed that environmental exposures can lead to DNA methylation of sperm. However, some research teams thought that generations cannot “inherit” this methylation change. They thought that the biological information carrier acting as the transmission medium is the small noncoding RNAs in sperm (sncRNAs) [94–96]. The RNAs do not encode protein and less than 50 nucleotides (nt), mainly including ribosomal RNA (rRNA), short interfering RNA (siRNA), microRNA, PIWI-interacting RNA (piRNA), and transfer RNA (tRNA). The expression of sncRNAs is abundant in mature sperm. These RNAs are regulated by paternal environmental conditions and can be transferred to the zygote during fertilization to regulate early embryonic development [96].

The epigenetic information in mammalian sperm can exist in the form of sncRNAs, which has attracted researchers' interest in recent years. With the development of research methods, people are skilled in using high-throughput technology, the research of RNA is more and more in-depth, and researchers pay close attention to various types of RNA in sperm. Altered sncRNAs of fertilized egg affect transmission of acquired phenotypes [51, 74, 75, 97]. In male mice under 6 weeks of chronic stress, the sensitivity of the offspring's hypothalamus-pituitary-adrenal (HPA) axis is blunted due to nine miRNAs expressions upregulated in the paternal sperm, and the blood corticosterone of the offspring is significantly reduced after acute stress [74]. Zygotic injection of the nine miRNAs reduce the stored maternal mRNA transcripts, including sirtuin 1 (Sirt1) and ubiquitin protein ligase E3a (UBE3A) [75]. UBE3A and Sirt1 play a nonnegligible role in the development of synapses and neuronal structure [98, 99]. The sncRNAs expression was abnormal in sperm of male mice which underwent early traumatic stress. Five microRNAs expressions were dysregulated in the blood and hippocampus of adult F1 and F2 offspring. After microinjection of total sperm RNAs from traumatized mice into oocytes of wild-type fertilized mice, the offspring showed depressive-like behavior [51]. One of these, miR-375 is involved in mediating the synthesis of catecholamines and negatively regulates the expression of tyrosine hydroxylase and dopamine-*β*-hydroxylase [100]. It is unclear how many generations of this behavioral abnormality mediated by sperm microRNAs can be inherited. Sperm tRNA-derived small RNAs (tsRNA) may be a paternal epigenetic factor. Injecting sperm tsRNA components from high-fat males into normal oosperm caused metabolic abnormalities in F1 offspring [76]. Male zebrafish exposed to chronic stress before mating resulted in the differential expression of 12 miRNA clusters, 6 piRNA clusters, and 12 tsRNA clusters in sperm. Most of the differentially expressed miRNA target genes are involved in mitochondria and Notch signaling pathway. The larva's ability to respond to dangerous environments is reduced by 2-3%, and the level of cortisol is elevated [77]. sncRNA could also mediate generational inheritance by altering histone modifications. Microinjection of miRNA-124 into single-cell embryos increased embryonic stem cell proliferation and modulated adult generations growth though increasing histone H3 methylation levels and modulating the transcriptional expression of Sox9. Levels of the miR-124 are not quantitatively altered in the embryos and adult offspring brain when compared to control mice [73]. Progeny characteristics can be inherited through the exposure or experience of ancestors, and certain paternal characteristics can be “remembered” in sperm through sncRNAs as information.

## 7. Future Prospects

Mature sperm deliver DNA, histone, chromatin-associated proteins, and sncRNAs to the oocyte at the time of fertilization, and hence potentially regulate early embryonic, fetal, and offspring development. The epigenetic landscape of sperm in response to various environmental conditions results in the transmission of paternally acquired phenotypes in offspring. The recent years have witnessed much progress in the area of environmentally induced epigenetic transgenerational inheritance of disease and phenotypic variation. However, the epigenetic landscape has grown increasingly complicated, and the mechanistic basis of such inheritance is only starting to be understood. Improving our understanding of epigenetic changes will enhance reproductive success and improve offspring health. Future studies will have to elucidate and clarify the role and underlying mechanisms of sperm epigenetics in those processes.

Although different epigenetic modifications, DNA methylation, histone modifications, sncRNAs, and sncRNAs-associated modification, have been identified, further researches are needed to explore the cross-links between these epigenetic processes and how they are regulated in normal development as well as diseased condition. DNA methylation, histone modification, and sncRNAs interact with each other [73, 101]. How the sperm microRNAs interact with the methylation and histone code throughout embryo development still needs more investigation.

Current epigenetic analyses are a multistep, high-cost research process. Low-cost and high-throughput techniques and tools should be developed. In addition, further exploratory researches are required to determine what epimutation patterns or signatures are associated with specific disease and/or ancestral exposures in humans and are necessary for the identification and clinical validation of sensitive and safe epigenetic biomarkers and drugs for an individual with specific disease susceptibility or environmental toxicant exposures.

## Figures and Tables

**Table 1:** Summary information of the relationship between environmental exposures, sperm epigenetic changes, genes, and offspring influence.

Species	Exposures	Epigenetic changes	Genes	Offspring influence	References
Human	War	Not mentioned	Not mentioned	Anxiety, depression, commit suicide	[9, 10]
Human	Smoking	Not mentioned	Not mentioned	Asthma	[11]
Human	Phthalate	Not mentioned	Not mentioned	The quality of blastocyst decreased	[12]
Human	Old	Not mentioned	Not mentioned	Autism	[13, 14]
Human	Drinking	Not mentioned	Not mentioned	Social psychological abnormality, neurocognitive development disorder	[15]
Rat	Endocrine disruptors	DNA methylation	LPLase	Fertility disorders	[66]
Mice	Nicotine		Dopamine receptors	Behavioral disorders	[67]
Mice	Early life stress		Protein kinase C	Cognitive impairment	[68]
Mice	Kdm1a overexpression	Histone modification	H3K4me3	Impaired development and survivability	[69, 70]
Rat	Cocaine		BDNF	Cocaine resistance phenotype	[71]
Rat	Hepatotoxin carbon tetrachloride		PPAR-*γ*	Suppressing fibrogenesis	[72]
Mice	Microinjection of miR-124 RNA in the one-cell embryo		Sox9	The “giant” phenotype	[73]
Mice	Chronic variable stress	Small noncoding RNAs	miR-193-5p, miR-204, miR-29c, miR-30a, miR-30c, miR-32, miR-375, miR-532–3p, miR-698	Reduced HPA stress axis responsivity	[74, 75]
Mice	Early traumatic stress		miR-375-3p, miR-375-5p, miR-200b-3p, miR-672-5p, and miR-466-5p	Depressive-like behavior	[51]
Mice	High-fat diet		m5C and m2 G in tsRNA fraction (30-40 nt)	Metabolic abnormalities	[76]
Zebrafish	Chronic stress		12 miRNA clusters, 6 piRNA clusters, and 12 tsRNA clusters	Reduced alertness to danger	[77]
